# Supplementary material for: Sounds are remapped across saccades
Source: Sci Rep. 2020 Dec 7;10:21332. doi: 10.1038/s41598-020-78163-y (PMC7721892; doi:10.1038/s41598-020-78163-y)
Supplement: Supplementary file 1 — Supplementary Figures. [file 41598_2020_78163_MOESM1_ESM.pdf]

Sounds are remapped across saccades

Martin Szinte[1*], David Aagten-Murphy[2], Donatas Jonikaitis[3], Luca Wollenberg[2,4] & Heiner Deubel[2]

1 Institut de Neurosciences de la Timone,
Centre National de la Recherche Scientifique, Aix-Marseille Université, UMR 7289, Marseille, France.
2 Allgemeine und Experimentelle Psychologie,
Ludwig-Maximilians-Universität München, Munich, Germany.
3 Department of Neurobiology and Howard Hughes Medical Institute,
Stanford University School of Medicine, Stanford, USA.
4 Graduate School of Systemic Neurosciences,
Ludwig-Maximilians-Universität München, Planegg-Martinsried, Germany.

*martin.szinte@gmail.com

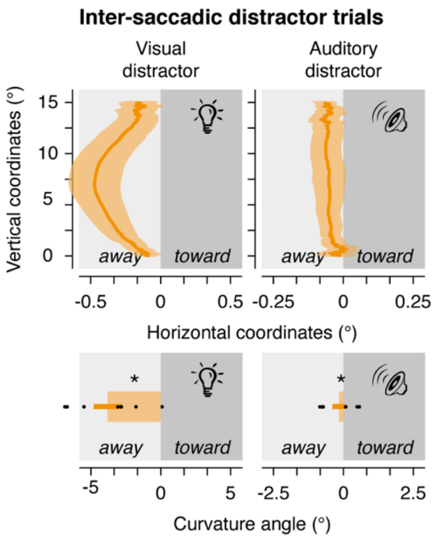

Figure S1. Inter-saccadic distractor trials. Averaged normalized second saccade trajectory (top panels) and curvature angle (bottom panels) observed following the presentation of a visual (left panels) or an auditory (right panels) inter-saccadic distractor in Exp. 2. Saccade trajectories are rotated in order to have upward saccades and negative x-values representing coordinates away from the screen position of the distractor. Areas around the averaged saccade trajectory and error bars represent SEM. Black dots show individual participants. Asterisk indicates a significant effect ($p < 0.05$, ns: non-significant).

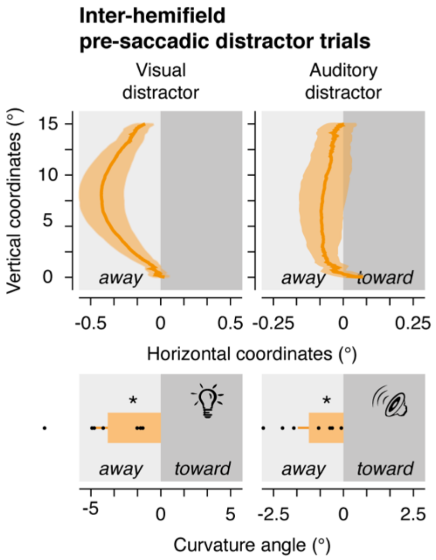

Figure S2. Inter-hemifield pre-saccadic distractor trials. Averaged normalized second saccade trajectory (top panels) and normalized curvature angle (bottom panels) observed following the presentation of a visual (left panels) or an auditory (right panels) inter-hemifield pre-saccadic distractor in Exp. 2. Conventions are as in Figure S1.
